# Patient with Herlyn–Werner–Wunderlich syndrome and endometriosis achieves successful full-term pregnancy (40 weeks and 6 days): a case report

**DOI:** 10.1186/s13256-024-04695-w

**Published:** 2024-08-03

**Authors:** Juliana Vieira Queiroz Almeida Oliveira, Chris Elizabeth Philip, Thayná Andreza Ribeiro Pereira, Gabriela Martins Perez Garcia, Quésia Tamara Mirante Ferreira Villamil

**Affiliations:** 1 Federal University of Minas Gerais, Belo Horizonte, Minas Gerais Brazil; 2 Department of Gynaecology, Beaumont Hospital, Dublin, Ireland; 3 Universidade Federal do Triângulo Mineiro, Uberaba, Minas Gerais Brazil; 4 Instituto Villamil, Belo Horizonte, Minas Gerais Brazil

**Keywords:** Müllerian ducts, Herlyn–Werner–Wunderlich syndrome, Obstructed hemivagina, ipsilateral renal anomaly, Pregnancy, Case reports

## Abstract

**Introduction:**

Herlyn–Werner–Wunderlich syndrome , a rare Müllerian ducts congenital disease, is characterized by a diphtheritic uterus, blind hemivagina, and ipsilateral renal agenesis. Diagnosis is at young age by ultrasound and magnetic resonance imaging, and the prognosis is good. Usually, complications evolve endometriosis and secondary pelvic inflammation.

**Case report:**

A 40-year-old female patient, Brazilian, white, primigravida, diagnosed at 30 years with a didelphic uterus on ultrasound, and 4 years later, with a left ovarian endometrioma, multiple ovarian cysts, and left renal agenesis on magnetic resonance imaging. Subsequently, due to dyspareunia and a feeling of swelling, the patient underwent transvaginal ultrasound with bowel preparation, and a hematocolpos was found and Herlyn–Werner–Wunderlich syndrome was suspected; 10 years after the diagnosis she had a planned pregnancy. She presented frequent contractions following the 15th week of pregnancy and fortunately there were no complications or premature labor. Labor was inducted at 40 weeks and 6 days without progress and a cesarean section was indicated and performed without complications. Herlyn–Werner–Wunderlich syndrome often goes unnoticed, leading to inadequate treatment. Individuals with Herlyn–Werner–Wunderlich syndrome commonly face fertility issues, such as high miscarriage rate (21–33%), and obstetric complications, such as spontaneous abortions (40% risk), intrauterine growth restriction, postpartum hemorrhage, increased fetal mortality, preterm delivery (21–29%), and elevated rates of cesarean sections. In addition, there is higher susceptibility of developing endometriosis, especially with hemivaginal obstruction, and pelvic adhesions.

**Conclusion:**

Early diagnosis enables timely treatment and, consequently, fewer complications. Still, when these factors are absent, vaginal birth may still be possible. The true prevalence and incidence of complications related to Herlyn–Werner–Wunderlich syndrome are still unknown.

## Introduction

Herlyn–Werner–Wunderlich Syndrome (HWWS) or obstructed hemivagina, ipsilateral renal anomaly (OHVIRA) syndrome, is an extremely rare congenital anomaly [1] affecting the female reproductive system, accounting for 5–11% of all Müllerian duct anomalies (MDAs) according to the American Society for Reproductive Medicine [2]. It is estimated that obstructed Müllerian agenesis affects 0.1–3.8% of women [1]. The exact incidence of OHVIRA in the general population is still unknown. It is characterized by uterus didelphys, a blind hemivagina, and ipsilateral renal agenesis. The exact cause of HWWS is still unknown, but it may be related to an abnormal development of the Müllerian and Wolffian ducts [3], making it the rarest form of Müllerian duct anomaly (MDA) (4.3–6.7%), with most cases being reported individually or in small series [4].

Diagnosing HWWS is often accomplished by using ultrasonography and magnetic resonance imaging (MRI). This can identify a uterus didelphys and a partial or complete duplication of the uterine, cervical, and vaginal structures. Treatment of HWWS usually involves resecting the vaginal septum, present in 75% of cases [5], at the time of menstruation when a large hematocolpos can be seen and palpated. The overall prognosis for HWWS is good and early diagnosis can help prevent secondary pelvic endometriosis and inflammation [3], as well as fertility and pregnancy complications in the future.

We present a case of a pregnant 40-year-old woman with multiple comorbidities, such as hemorrhoids and endometriosis, who had a late diagnosis of HWWS at the age of 38 years. She successfully carried her pregnancy to term, as desired by her, with a very favorable outcome. Ethical committee approval number: 70976823.4.0000.5105.

## Case report

A 40-year-old, primigravida woman, white, weighing 60 kg with a history of HWWS was seen in the hospital for her pregnancy.

**Fig. 1:**
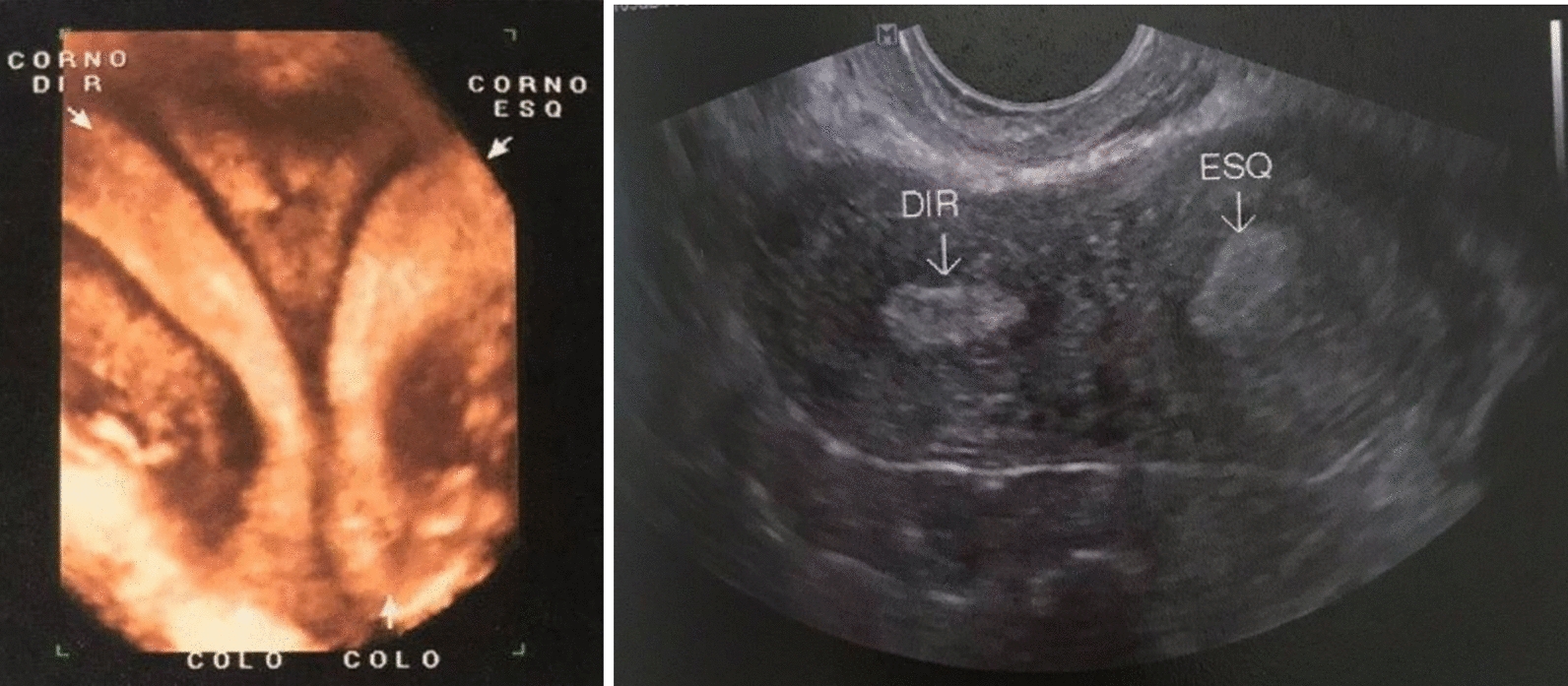
Ultrasound showing the didelphys uterus at diagnosis. In the image on the left, the right (“Corno dir”) and left horns (“Corno esq”) can be visualized, as well as the two cervical canals (“colo”) of the patient, marked in the lower region of the image. On the right, we can see the didelphic uterus, with arrows pointing to the right (“DIR”) and left (“ESQ”) uteri

She was diagnosed with a didelphys uterus measuring 8.0 cm × 6.3 cm × 2.9 cm with a volume of 65 cm^3^ after she expressed concerns about a possible miscarriage when she was 30 years old for an unusually heavy hemorrhagic episode (Fig. 1); 4 years later, an MRI revealed a left ovarian endometrioma measuring 1.6 cm × 1.1 cm, multiple ovarian cysts ranging from 0.3 to 1.3 cm in size, and a left-sided renal agenesis. A transvaginal ultrasound scan (TVS) confirmed a 1.1 cm × 1.1 cm endometrioma in the left ovary, indicating endometriosis in the left uterus-sacral ligament and showing partial stenosis of the external orifice of the left cervix with cervical mucus and pelvic adhesions. The left ovary was adhered to the uterus, resulting in reduced mobility of the uterus and its attachments. She was started on dienogest and continued with the medication until she decided to have a planned pregnancy.

A new MRI 2 years later showed a uterus with a left component measuring 40 cm^3^ and a right component measuring 35 cm^3^. Additionally, a nabothian cyst was identified in the uterine cervix along with multiple small ovarian cysts and a leiomyoma in the anterior wall of the left component, and 2 months later a TVS was performed to map her endometriosis, which showed some echographic findings suggestive of nonspecific endometriosis in the iliac fossa. The TVS also identified a painful tissue scar near the transducer between the cervix and vagina, an expansive lesion in the cervix, and no signs of adenomyosis.

After a period of 2 months, another follow-up TVS was conducted with bowel preparation in response to complaints of dyspareunia and a sensation of vaginal swelling. The TVS this time revealed a left uterine component measuring 25 cm^3^ and a right component measuring 20 cm^3^. Within the wall of the left uterine cervix, a cystic formation was detected that was heterogeneous in nature and mobile. It also exhibited thin walls and had no flow on Doppler, measuring 2.6 cm × 2 cm × 2.1 cm. This cystic formation was found to be painful upon direct compression with the transducer. Given the presence of hematocolpos and considering the patient’s medical history, the possibility of Herlyn–Werner–Wunderlich Syndrome was suggested due to the presence of a didelphic uterus, renal agenesis, and a history of a spontaneously ruptured hemivaginal septum during adolescence.

**Fig. 2:**
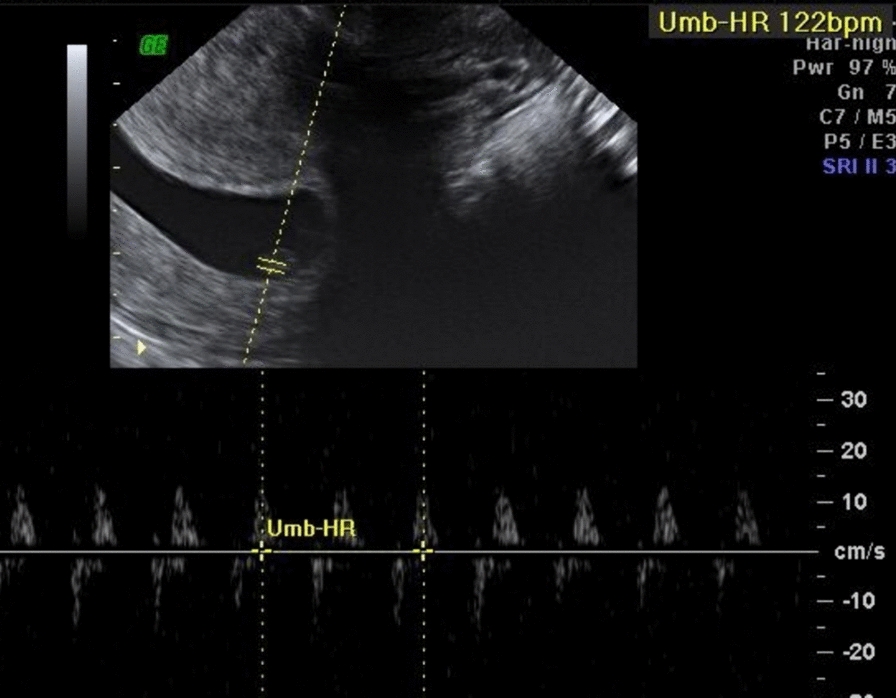
Ultrasound showing presence of fetal heartbeat in one uterus

Then 10 years after being diagnosed with a didelphys uterus, she decided to have a planned pregnancy. At the time under consideration, she was 40 years old, and her partner was 42 years old, and 2 months after initiating prenatal multivitamin supplementation and discontinuing her contraceptive use, an early ultrasound revealed a pregnancy at 6 weeks and 1 day of gestation, and confirmed the presence of fetal heartbeats (Fig. 2). She began physical therapy with a specialist at a women’s health clinic, aiming to enhance pelvic stability and resilience to better accommodate the physiological demands of pregnancy. However, at 15 + 2 weeks she started having contractions and was started on oral progesterone 200 mg twice a day, and this treatment continued until she reached full term. During this period, she also suffered from headaches, general distress, abdominal swelling, and lower abdominal pain, which worsened with physical activity. She was also noted to have a rectus abdominis diastasis of 1.5 cm.

At 24 + 5 weeks she started experiencing a pain in her right vulva where varices were observed and she was also noted to have hemorrhoids. She was put on compressive stockings and prescribed a cream for her varices and hemorrhoids. Her supraumbilical diastasis increased to 3.5 cm with no projection during trunk flexion.

Routine ultrasound examinations showed a posterior placenta with a normal anatomy scan along with normal Doppler findings. At 27 + 3 weeks she was recommended bed rest due to a threat of premature labor (softened cervix and uterine contractions that eased with rest), and she continued to work at home. At 32 + 4 weeks, she started experiencing regular uterine contractions 30 minutes apart but without vaginal bleeding or cervical dilation. She was advised to stop working at home, due to stress, and continue with her bed rest. She continued experiencing irregular frequent contractions and continued using progesterone until she reached 37 weeks. Through this time, she continuously experienced pains during exercise, vulval pressure, hemorrhoids, and redness in her lower limbs. Since the patient preferred and insisted on a vaginal birth, labor induction was not considered until 40 weeks and 6 days of gestational age. Through this period, she attended weekly appointments with both her obstetrician and physical therapist, with strict instruction to return to the hospital if there was a change in baby movement or a natural progression of labor.

At 40 weeks and 6 days, surprisingly, despite these ongoing contractions, her labor still required induction. Misoprostol as well as a mechanical dilator of balloons were used but they fell out. An epidural was requested at 7 cm dilation and oxytocin was commenced alongside with it. She progressed to full dilation and 90% effacement as expected, however, it was noted that fetal descent stopped. A non-reassuring Cardiotocography (CTG) forced an attempted forceps delivery but was unsuccessful and it was converted to an emergency caesarean section, despite the physician’s extensive experience in vaginal births and forceps use. A healthy baby boy, weighting 3560 g, was delivered with no complications noted to the mother or baby.

During follow-up visits, the patient was breastfeeding and reported moderate pain in her surgical would. She also had a small hematoma (2 × 1 cm) on her vulva, which increased to 4 cm over the next week. The hematoma was subsequently drained and treated with cephalexin, and 40 days post-caesarean, the patient had no complaints and her surgical wound was healing well. There were no additional complications.

## Discussion

HWWS presents clinically as dysmenorrhea, abdominal pain, pelvic mass, and foul mucopurulent discharge. Often, it goes unnoticed or is misdiagnosed, leading to inadequate treatment due to its uncommon and diverse clinical manifestations. Individuals with HWWS commonly face fertility issues, including a higher miscarriage rate (21–33%) compared with the general population (15–20%) [6], as well as obstetric complications such as intrauterine growth restriction, abnormal fetal presentation, postpartum hemorrhage, premature rupture of membranes, increased fetal mortality, and elevated rates of cesarean sections [7]. Those with HWWS are also more susceptible to developing endometriosis and pelvic adhesions, especially when a unilateral vagina is obstructed. Accumulated materials within the uterine cavity can also lead to infections [5].

In 2015, a novel classification was suggested by Zhu, which differentiated complete (30%) and incomplete (70%) obstructed hemivagina on the basis of their distinct clinical presentations [3]. Complete obstructions tend to exhibit symptoms at an earlier age (12.86 ± 1.84 years) and are more frequently linked to endometriosis, hematometra, hematosalpinx, hemoperitoneum, pyosalpinx, and pyocolpos compared with incomplete obstructions [3].

Endometriosis and pelvic infections are also commonly noted complications. In the reported cases of pelvic endometriosis or adhesions it is noted that endometriosis and its occurrence was higher in cases of complete hemivaginal obstruction [12] Removal of endometriomas before pregnancy is recommended in cases with long-term progression due to the stronger association between infertility, endometriosis, and pelvic infections rather than uterine anatomical anomalies.

Complications regarding pregnancy in HWWS are primarily associated with a 40% risk of spontaneous abortions, with a greater risk observed following surgical vaginal septum correction. During pregnancy, complications mainly revolve around preterm deliveries (ranging from 21% to 29% versus 9% to 10% in the general population) [11]. Diagnosis of pelvic anomalies before pregnancy is crucial for achieving successful reproductive outcomes [11].

A retrospective study revealed only 6 out of 70 patients were diagnosed with primary infertility postoperatively, indicating a normal incidence of primary infertility in women with HWWS [9]. In this patient’s case, she had not undergone surgery, lacked a definite history of proven miscarriages, and despite facing preterm delivery risks, successfully carried a full-term pregnancy.

The connection between HWWS and mode of delivery lacks definitive establishment, though vaginal delivery is not explicitly contraindicated. Reports mention cases of vaginal birth with the need for mediolateral episiotomy [10], while others do not specify the need for intervention during delivery. A retrospective study covering 94 cases over 20 years indicated a 75% cesarean section rate, with 47% attributed to malposition (breech or transverse) [13]. Presently, no recommendations exist for the mode of delivery in cases of didelphys uterus [13].

We also found that in documented cases of HWWS, in regards to gestational age (GA) at birth, it is not uncommon for term labor to be observed, with cases reported ranging from 36 weeks [1] to 39 weeks and 6 days [10]. Our patient represents the longest GA that will be found in literature to date.

Existing literature indicates a higher incidence of right-sided renal agenesis (60%) [4], which differs from the presented case of left renal agenesis. The patient did not exhibit typical symptoms and her diagnosis was finally made after years of follow-up scans of an incidental finding at the age of 37 years, falling within the typical age range of HWWS diagnosis (10–50 years) [8, 9]. A retrospective study also noted a high rate of contralateral gestation (63.5%) [9], which differs from our patient’s case involving pregnancy in the same uterus as the vaginal septum.

Timely correction of malformations and lesions leads to improved reproductive outcomes, allowing both uteri to support normal pregnancy and subsequent delivery. While fertility remains intact and fetal survival rates are high, concerns remain regarding premature rupture of membranes and delayed fetal development [14]. Therefore, when a unilateral renal agenesis and uterus didelphys coexists, the presence of a blind vagina must be ruled out first [15].

## Conclusion

We emphasize the significance of early diagnosis in HWWS cases, as it enables timely treatment and disease management, leading to fewer complications such as endometriosis and pelvic adhesions, which are known to be associated with abortion and infertility. However, the scarcity of reports in the literature concerning HWWS and pregnancy makes it challenging to determine the true prevalence and incidence of complications in women with this condition.

## Data Availability

Data cannot be shared openly due to protection of the study participant privacy.
